# SPECT/CT Correlation in the Diagnosis of Unilateral Condilar Hyperplasia

**DOI:** 10.3390/diagnostics11030477

**Published:** 2021-03-08

**Authors:** Diego Fernando López, Valentina Ríos Borrás, Juan Manuel Muñoz, Rodrigo Cardenas-Perilla, Luis Eduardo Almeida

**Affiliations:** 1 Orthodontics Department, Universidad del Valle, Cali 760043, Colombia; 2 Escuela de Odontología, Universidad del Valle, Cali 760043, Colombia; valentina.rios@correounivalle.edu.co; 3 Nuclear Medicine Department, Centro Médico Imbanaco, Cali 760043, Colombia; juan.munoz@imbanaco.com.co (J.M.M.); rodrigo.cardenas@imbanaco.com.co (R.C.-P.); 4 Surgical Clinical Sciences, School of Dentistry, Marquette University, Milwaukee, WI 53206, USA; luis.almeida@marquette.edu

**Keywords:** bone scintigraphy, computed tomography, condylar hyperplasia, SPECT, ^99m^Tc-MDP

## Abstract

Objective: To evaluate the correlation between metabolic bone activity measured by single photon emission computed tomography (SPECT) and the anatomic condylar characteristics acquired by computed tomography (CT), in patients with unilateral condylar hyperplasia (UCH). Method and Materials/Patients: Observational, descriptive study in a group of 71 patients with clinical diagnosis of UCH and indication of SPECT/CT. Bone SPECT images obtained in a gamma-camera GE Infina and processed in a station Xeleris 3 with the program Volumetrix MI Evolution for bone. CT images acquired in a PET/CT Biograph mcT20 equipment (Siemens) processed in a station Osirix V 7.5.1 (Pixmeo, Bomex, Switzerland). Results: The sample included 24 men (33.8%) and 47 women (66.2%). Active state UCH was detected in 40 (56.3%) cases (over 55% uptake in the affected condyle) and 38 (53.5%) presented mandibular deviation to the right side. No significant differences related to sex, age, or mandibular deviation side were found. Mandibular deviation was the only morphologic feature related to active/inactive UCH (*p* = 0.003). The likelihood of active CH was significantly higher in patients with mandibular deviation higher than 6 mm compared with <6 mm (odds ratio (OR): 3.51, confidence interval (CI) 95%: 1.27–9.72). Conclusion: There is a significant correlation between the magnitude of mandibular deviation quantified on CT and metabolic findings obtained by SPECT in patients with UCH. The risk of active UCH is 3.5 times higher in patients with a mandibular deviation ≥6 mm.

## 1. Introduction

Condylar hyperplasia (CH) is a progressive and self-limiting pathology affecting the mandibular condyle growth and compromising the temporomandibular joint (TMJ) anatomy [1,2,3].

The functional, occlusal, and esthetic effects of CH in patients demand a multidisciplinary intervention to confirm a clinically suspected diagnosis and establish the therapeutic approach [4]. Early diagnosis and adequate treatment are important to avoid complicated sequelae [5].

UCH is effectively diagnosed by measurement of bone metabolic hyperactivity in SPECT mandibular TMJ 3D images [1,6,7]. Recent studies show that 3D SPECT images are superior to planar images [8]. Uptake radioactive values equal or higher than 55% for the suspected condyle or a percentage side difference over 10% are commonly accepted as positive results indicating hyperactivity (active disease) of the mandibular condyle [9,10]. However, the functional SPECT images are not adequate to show in detail the anatomic structures in the region of interest (ROI). Therefore, it is recommended to combine SPECT with CT images to characterize the pathology by both its anatomy and metabolism [11,12].

The correlation of metabolic and anatomic findings by a team of experts in the management and interpretation of SPECT/CT techniques allows the establishment of clear-cut parameters of the pathology to precisely indicate the extension of the altered region. This approach is a recent breakthrough in the procedures of diagnosis and treatment of UCH [12,13].

The authors’ hypothesis from the existing literature is that the fusion of images and information obtained from SPECT/CT to diagnose UCH improves the precision and specificity of the diagnostic tests and, consequently, allows better therapeutic decisions [11,12,14,15,16]. Considering that there are few published studies and they provide information from poorly representative populations, the objective of the present study was to correlate the metabolic bone activity of the condyle measured by SPECT with the anatomic information provided by CT images, in patients with active or inactive UCH.

## 2. Materials and Methods/Patients

This is a retrospective observational study with no intervention or manipulation of variables from the patients. Therefore, it is a no risk investigation and was approved by the Institutional Ethics Committee (Approval number CEI-403) and conducted following all the regulations of the Declaration of Helsinki, last version.

**Figure 1:**
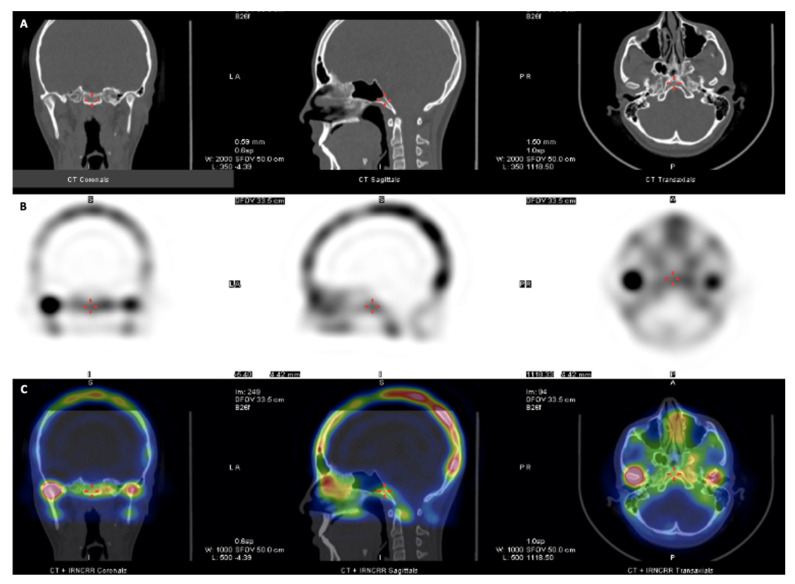
(**A**) Computed tomography (CT) images: coronal, sagittal, and axial sections. (**B**) Single photon emission CT (SPECT) images: coronal, sagittal, and axial sections. (**C**) Fused functional images (SPECT and CT) in a patient with right side active unilateral condylar hyperplasia (UCH).

A population of 153 image sets from patients tested by SPECT/CT (Figure 1), performed in the Nuclear Medicine deparment of a High Complexity Center, between January 2015 and January 2020, was evaluated for the study. The patients had been sent to Nuclear Medicine by the clinical specialists owing to facial asymmetry and suspected UCH. Following the classification of Lopez et al. 2019 [17], the patients were classified by types of facial asymmetry, obtaining 71 cases with UCH diagnosis. Taking into account the information from clinical records, patients with antecedents of TMJ trauma or fracture, previous orthognatic surgery, dentofacial syndromes, and arthritis were excluded. When the SPECT/CT information was not complete, the set of images was excluded as well.

The mandibular bone SPECT procedure was carried out 2 h after the endovenous administration of a dose of 15 mCi ^99m^Tc-MDP for patients over 18 year and normalized according to the EANM Pediatric Dosage Card for patients under 18. The images were obtained using a double head gamma-camera GE Infinia (Chicago, IL, USA), with low energy collimators, and a 128 × 128 high resolution matrix to obtain 45 images, 18 s of exposure, for each 180° of detection.

The data were reconstructed in the processing station Xeleris 3 (General Electric, Chicago, IL, USA) using the program “Volumetrix MI Evolution for Bone”, with the ordered subsets expectations maximization (OSEM) algorithm for iterative reconstruction, applying four interactions and eight subsets with a Butterworth 0.45 filter and Power 12, plus correction of resolution recovery. From the reconstruction, five transaxial images for quantification in the condyles were obtained, extracting the total counts for a fixed-size ROI (1.76 cm^2^) [18].

The SPECT report provided quantitative information expressed as radionuclide percentage uptake in the condyles. The counts observed within the selected ROI were used to calculate the % uptake using the following equations:

% right condyle uptake = Maximum counts in the right condyle × 100

Right side counts + left side counts

% left condyle uptake = Maximum counts in the left condyle × 100.

Right side counts + left side counts

A difference in percentage uptake between condyles = or >10% was interpreted as a positive result indicating active pathology [3].

CT cranial images were acquired in a PET/CT Biograph mCT20 (Siemens, Erlangen, Germany) equipment without contrast enhanced, from vertex to sternal notch, applying the following parameters: section thickness: 1.0 mm, pitch: 1.0, and a 512 × 512 cubic matrix with isotropic voxel (size 0.75 × 0.75 × 0.75 mm) to avoid image distortion in the different planes. The same parameters were applied to adult and pediatric patients. The CT images were reconstructed using a B26F homogeneous low dose filter for anatomic localization. All the patients were positioned with a head fixing device to avoid artifacts owing to movement and facilitate the image fusion.

The set of DICOM images was processed in a work station Osirix V 7.5.1 (Pixmeo, Bernex, Switzerland) obtaining linear measurements in sagittal and frontal planes. The description of measurements taken in the 3D bone tissue reconstruction is described in Table 1.

The tomographic measurements were taken by a trained and calibrated operator. Each data set was simultaneously revised and classified according to the craniofacial characteristics of the asymmetry [17], together by the operator and a specialist with experience in diagnosis and treatment of patients with facial asymmetry.

To assess the reproducibility of the measurements, a duplicate reading was taken by the same observer on a subsample of 20 patients with a four-week interval between the two measurements. The correlation coefficients (Rho) indicate an agreement higher than 0.90 for all of the variables (Table 2).

### Statistical Analysis

The data were processed by one operator expert in the software management. All statistical analyses were carried out in the software Stata13^®^ (StataCorp, College Station, TX, USA). Normality of distribution was tested by the method of Shapiro–Wilk and, according to it and the kind of variable, the results are expressed as average ± standard deviation, median, inter-quartile range, and absolute/relative frequencies. The Chi square test or Fisher test was applied for bivariate analysis of qualitative variables and either Student’s t-test or U-test for quantitative variables, according to the distribution normality. Correlations were evaluated by the Spearman coefficient rho. The level of significance was *p* < 0.05.

Intraobserver agreement was evaluated by the intraclass correlation coefficient of Lin.

Receiver operating characteristics (ROC) curves were determined to establish the best cut-off value of mandibular deviation to classify the hyperplasia as active or inactive. ROC curves were obtained from estimated sensitivity, specificity, and positive and negative predictive values calculating their 95% confidence intervals.

## 3. Results

Data from 71 SPECT/CT files were analyzed. The sample included 47 (66.2%) women and 24 (33.8%) men, with a median age of 19 years. From the total number of patients, 40 (56.3%) presented active UCH (≥55% uptake in the affected condyle) and 38 (53.5%) presented right side deviation. No significant differences in the frequency of active UCH were detected in relation to age, (*p* = 0.1), sex (*p* = 0.22), or side of mandibular deviation (*p* = 0.99) (Table 3).

### Morphologic Data and Active Hyperplasia

**Figure 5:**
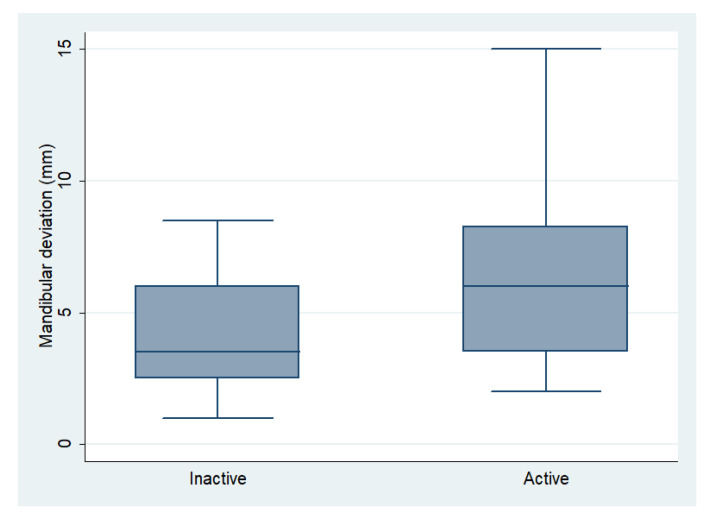
Mandibular deviation 95% confidence intervals (CIs) in active and inactive UCH patients.

The measurements obtained in CT images of the patients were related to the active or inactive state of UCH. The results are presented in Table 4 and Figure 5. A statistically significant difference was found only for the amount of mandibular deviation, which was higher in active cases of UCH (6.3 ± 3.4 mm) compared with inactive cases (4.1 ± 2.2) (*p* = 0.003).

**Figure 6:**
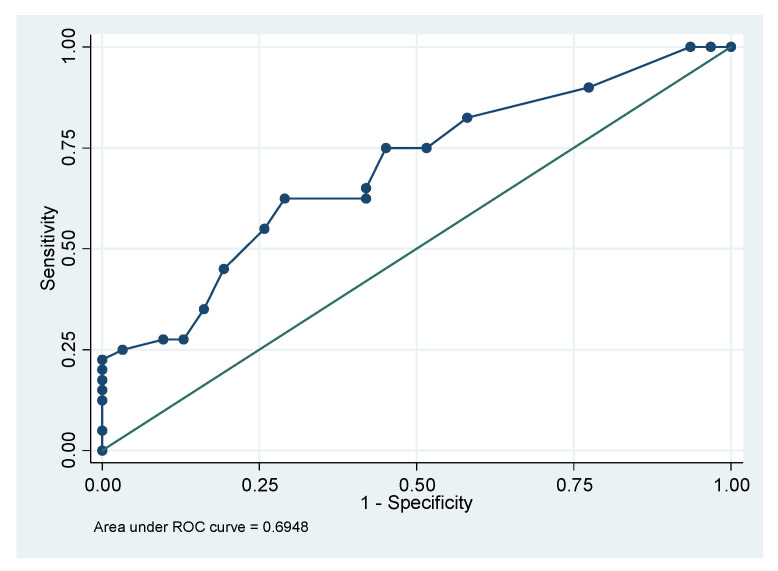
Receiver operating characteristics (ROC) curve (sensitivity vs. 1—specificity) for condylar hyperplasia activity detection by mandibular deviation.

The ability of mandibular deviation to classify the state of UCH as active or inactive was studied by ROC analysis. The area under ROC curve (AUC) was 0.695 (CI 95%: 0.57–0.82), indicating acceptable ability to distinguish the states, as the area is >0.5 (Figure 6).

Two cut-off values of mandibular deviation were selected. The first was a 6 mm value because it is more specific, that is, it detects inactive UCH with 55% sensitivity and 74.19% specificity, providing a positive predictive value (PPV) of 73.3% and negative predictive value (NPV) of 56.1%. The other cut-off value was 4 mm, which shows the best sensitivity, that is, it detects more active cases of UCH with a sensitivity of 75% and 54.8% specificity, PPV of 68–18%, and NPV of 63% (Table 5).

The likelihood of having active UCH in patients with mandibular deviation equal or higher than 6 mm was 3.5× higher than the likelihood associated with mandibular deviations <6 mm (OR: 3.51, CI 95%: 1.27–9.72).

When the cut-off value was set to 4 mm, the likelihood of inactive UCH was 73% (OR: 0.27, CI 95%: 0.10–0.75).

## 4. Discussion

Image fusion for diagnostic purposes, as in the case of SPECT/CT, is known as a co-register or hybrid technique and it is used to improve the diagnostic precision and, therefore, to aid in the development of a better treatment plan positively determined by the prognosis [19]. In nuclear medicine, the use of hybrid tests increases the diagnostic precision by about 30% in skeletal conditions, as well as in tumors and inflammatory processes, owing to a better correction of attenuation, higher specificity, and a more accurate description of the disease location and possible compromise of the adjacent tissues [20,21].

In connection with this, Jacene et al. [11] postulated that the hybrid SPECT/CT image compared with SPECT alone provides additional interpretative information because the CT data indicate the anatomic location of abnormal findings.

The radioactive uptake in bone SPECT depends on the blood circulation and the absorption by the structure of hydroxyapatite crystals. The areas of high uptake of the radioactive tracer are correlated with hyperemia and more metabolic bone activity and, additionally, identify activity at the molecular level. Therefore, nuclear medicine images are highly sensitive for early detection of lesions, very much earlier than X-ray or tomographic images. Bone SPECT is very useful and has been validated for early diagnosis of UCH [1,6,7,22], because this is a condition that could be active during growth and development, but may be self-limited and finally expressed only by sequelae of the pathology [2,23]. Although the diagnosis is strictly clinical, based on intraoral and extraoral findings and tomographic or radiographic images, the evaluation of bone metabolism by SPECT is very useful to differentiate the active/inactive stages [17].

The hybrid SPECT/CT technique for the diagnosis of UCH provides detailed morphologic information about the mandibular condyles and other craniofacial structures that may be compromised in the pathology. This information is associated with the data of bone metabolic activity in the condyles [12], obtained by the comparative lateral uptake of ^99m^Tc-MPD. In this context, Suh et al. [24] point out the need to have a standardized value for the radiopharmaceutical uptake and the CT data to evaluate temporomandibular disorders.

In the present study, the fusion of data from SPECT/CT to classify UCH conditions as active or inactive detected a significant difference in the magnitude of mandibular deviation (MD) associated with active cases. This finding is concordant with the results of Wang et al. [25], postulating that only MDs exceeding 5 mm are unacceptable according to the patient perception and demand for surgical treatment.

Regarding the diagnostic added value of SPECT/CT compared with SPECT alone to evaluate UCH, Fokoue et al. [26] indicate that this image fusion is superior to detect the hyperplasic area. Agarwal et al. [15] also evaluated, in 21 patients, the diagnostic improvement obtained by the SPECT/TCT fusion compared with SPECT alone, which is more sensitive (80%), but SPECT/CT is more specific (100%) and accurate (85.5%), while planar scintigraphy had the lowest diagnostic performance. However, Theerakulpisut et al. [14], in a study of 61 scintigraphies, concluded that the diagnostic specificity is not improved by fused tests and, as the radiation is increased, did not recommend its use. In the same sense, Verhelst et al. [27] reported that the anatomic changes detected by CT in the hybrid test are evident only in 50% of the patients, adding a minimum benefit, and Liu et al. [16] concluded that ROI delimitation in the drawing of condylar outline was not superior when SPECT/CT was used.

Taking into account these observations, the authors of the present study postulate that the specificity of the SPECT test is improved by the clinical and tomographic pre-diagnostic findings [17], and by the technique applied. The ROI selection; the number of trans-axial sections; and the quantification of radioactive uptake, either by total counts or mean counts, are critical aspects having an influence on the results of the test [18].

International studies indicate that, in different populations, the prevalence of UCH is higher in women than in men [13], as was found in the present study (66.2% women). However, the difference in number of active/inactive cases of the pathology was not significantly sex-dependent. Additionally, in the present study, the difference in laterognathia was not statistically significant, in agreement with previous reports [1,28].

Regarding the age distribution of UCH, the average age in the active UCH group was similar to that of inactive UCH (19 and 17, respectively), both including ranges of residual growth [29]. Although the early detection of UCH reduces the sequelae and invasiveness of the treatment, the fact that that only 10% of changes in bone metabolism appear as positive uptake in SPECT deserves consideration, but the anatomic changes detected by CT are able to indicate the compromise of a higher percentage of bone density [22]. Therefore, in very young patients or in patients that at the moment of examination have initial development of the pathology, the SPECT/CT correlation may not be positive because the pathology is not sufficiently expressed.

The difference between the average DM (6.3 mm) in active states of UCH and the average for inactive conditions (4.1 mm) is statistically significant (*p* = 0.003) and clinically relevant.

Therefore, a significant outcome of the study is the demonstration that a mandibular deviation >6 mm is able to classify the UCH condition as active or inactive, because the AUC in the ROC curve was 0.695. López et al. [30] recently evaluated the ability of mandibular deviation to differentiate the hemi-mandibular elongation (the most common form of condylar hyperplasia [17]) from the asymmetric mandibular prognathism, determining that MDs >5.1 mm are more frequent in hemi-mandibular elongation cases.

The present study provides data from a sample higher than other studies published to study the correlation SPECT/CT in UCH patients. However, a limitation of the study is that hybrid equipment was not used, but rather image fusion. There is no correction for attenuation in this case. Additionally, the use of two separate techniques generates more radiation and is more expensive than the SPECT alone, but the hybrid special scanning system is not yet available in development countries, except in a limited number of research institutions. It is also important to mention that volumetric assessment of the mandible and the articular surfaces provides information on the entire structure under study [31]; although, for this research, what was taken into account were linear measurements, including those of the active condylar surfaces such as the medial–lateral pole and the anterior–posterior pole, which represent the functional area, it is recommended that volumetric assessment of the articular structures be carried out in future studies.

## 5. Conclusions

The correlation between the magnitude of mandibular deviation measured in CT images and the percentage uptake obtained by SPECT is statistically significant (*p* = 0.003) and ROC analysis established that a mandibular deviation >6 mm is a risk factor for active UCH (OR: 3.51; CI 95%: 1.27–9.72).

## Figures and Tables

**Figure 2:**
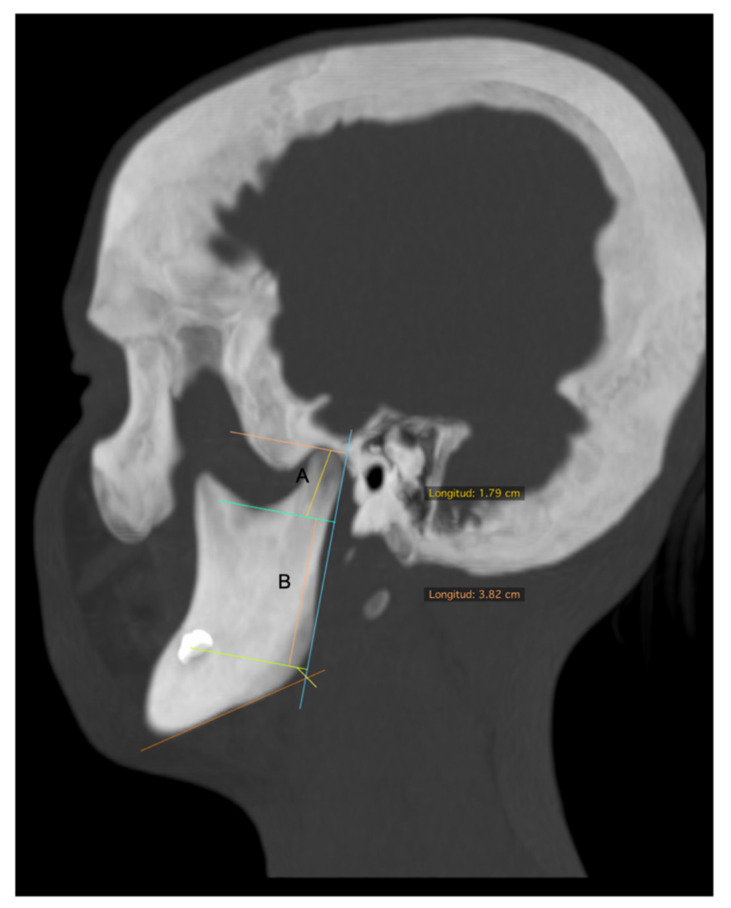
Bone tissue CT, sagittal view. (**A**) Condylar length. (**B**) Mandibular ramus length.

**Figure 3:**
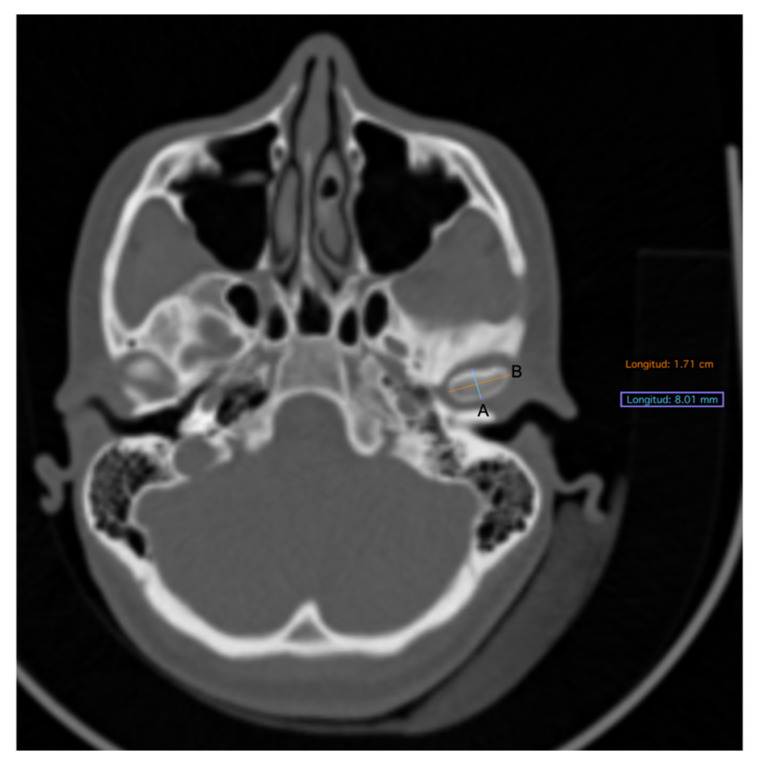
Bone tissue CT, axial view. (**A**) Anteroposterior condylar length. (**B**) Midlateral condylar length.

**Figure 4:**
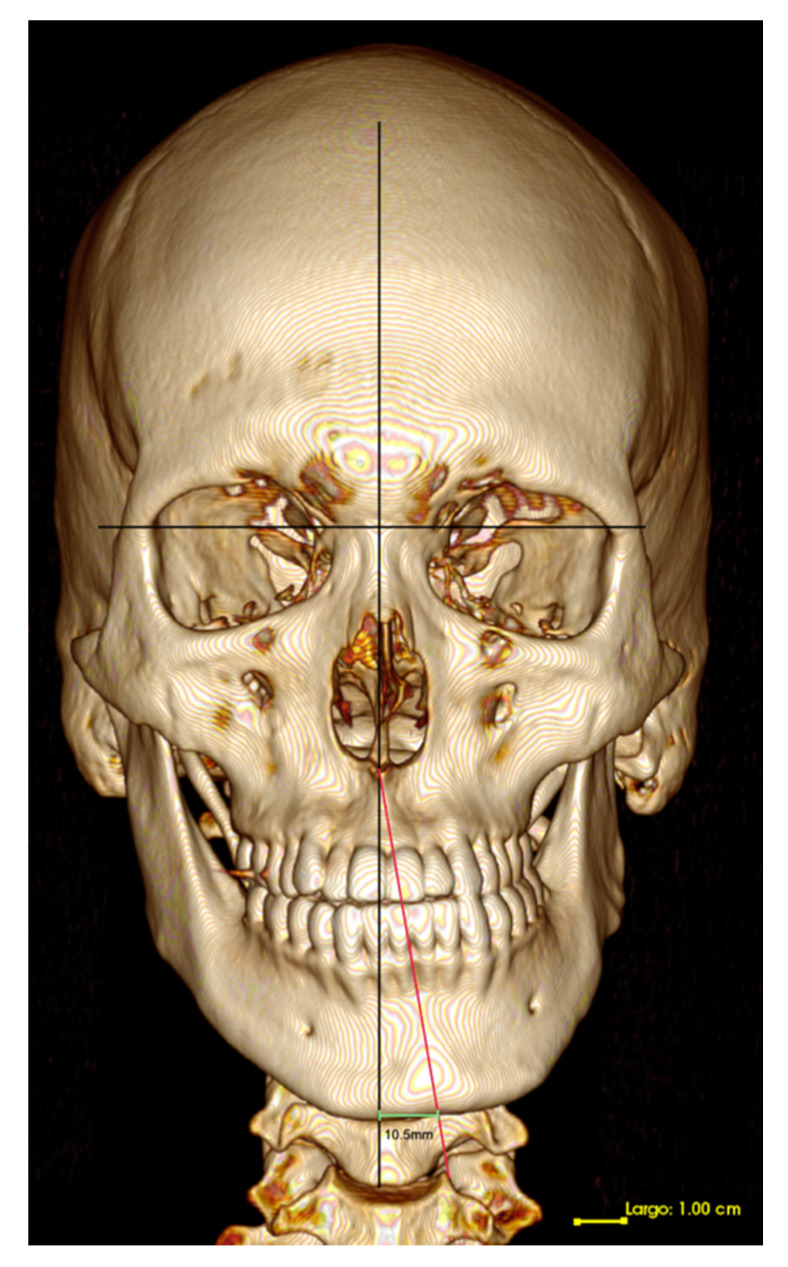
Bone tissue CT, 3D reconstruction. Coronal view. Mandibular deviation magnitude and deviation side.

**Table 1:** Description of craniofacial measurements taken in the 3D bone tissue reconstruction, frontal, and sagittal planes.

Measurement (mm)	Description
Condylar length	In the sagittal view, a line parallel to a tangent to the posterior ridge of the mandibular ramus was traced and extended from the most superior point of the condyle to a perpendicular line, passing through the most inferior point of the mandibular notch. This length was obtained in a corrected image of the axial axis of the mandibular ramus (Figure 2A).
Mandibular ramus length	In the sagittal view of 3D reconstruction, a line perpendicular to the Frankfort plane, was traced and extended from the deepest point of the notch to the inferior ridge of mandibular body (Figure 2B).
Anteroposterior condylar length	In the axial view, a line from the most anterior point of condylar cortical bone to the most posterior limit of cortical bone was traced. This image was obtained in an orthogonal plane (Figure 3A).
Midlateral condylar length	In the axial view, a line from the most anterior limit of proximal cortical of the condyle to the most anterior limit of its distal cortical was traced. This image was obtained in an orthogonal plane (Figure 3B).
Deviation Magnitude	In the coronal view of the 3D reconstruction, repositioning the skull in a natural position of the head, the magnitude of the deviation is quantified as follows: the distance in mm from menton to the facial midline projected from the apophysis *crista galli*, perpendicular to the zygomatic plane was measured (Figure 4).
Side of mandibular deviation (laterognathism)	In the coronal view of the bone tissue 3D reconstruction, this qualitative variable was visually detected, indicating the mandibular deviation side (right/left) (Figure 4).

**Table 2:** Intraobserver agreement for craniofacial measurements obtained in frontal and sagittal views.

Measurement in mm	Difference * Average ± SD	Rho	*p*-Value
Right side total condylar length	0.01 ± 0.19	0.99 (0.99–1.00)	0.000
Left side total condylar length	0.10 ± 0.31	0.99 (0.99–1.00)	0.000
Right side ramus length	−0.19 ± 0.33	0.99 (0.99–1.00)	0.000
Left side ramus length	0.10 ± 0.57	0.99 (0.99–1.00)	0.000
Anteroposterior pole of right condyle	0.07 ± 0.19	0.98 (0.96–0.99)	0.000
Anteroposterior pole of left condyle	0.19 ± 0.28	0.95 (0.91–0.99)	0.000
Lateral pole of right condyle	−0.19 ± 0.31	0.99 (0.98–1.00)	0.000
Lateral pole of left condyle	−0.05 ± 0.30	0.99 (−0.99–1.00)	0.000
Mandibular deviation in mm	0.13 ± 0.51	0.99 (0.98–1.00)	0.000
Difference in percentage uptake	0 ± 0	1	0.000

Difference between first and second measurement *. SD: standard deviation. Rho: Spearman correlation coefficient.

**Table 3:** General characteristics of the patients with active/inactive condylar hyperplasia. SPECT, single photon emission computed tomography.

Variable	Active *n* = 40	Inactive *n* = 31	Total *n* = 71	*p*-Value
Age	19 (16–26.25)	17 (13–21.5)	19 (15–25.5)	0.10
	22.15 (8.54)	19.29 (7.47)	20.90 (8.16)	0.14
Male sex	11 (27.5)	13 (41.93)	24 (33.80)	0.22
Female sex	29 (72.5)	18 (58.06)	47 (66.20)	
Right Laterognathism	21 (52.5)	17 (54.84)	38 (53.52)	0.99
Left Laterognathism	19 (47.5)	14 (45.16)	33 (46.48)	
Difference in percentage uptake in SPECT *	17.2 (7.52)	3.93 (3.16)	11.41 (8.93)	<0.001

Median (P25–P75); *n* (%); average (standard deviation). * (≥10% active stage).

**Table 4:** Comparison of morphologic measurements in active/inactive unilateral condylar hyperplasia (UCH) cases.

Morphologic Measurements in mm	Active	Inactive	*p*-Value
Right condyle total length	19.75 (3.41)	20.21 (3.07)	0.55
Left condyle total length	19.95 (4.48)	19.53 (4.88)	0.71
Right mandibular ramus length	34.96 (5.0)	37.29 (6.37)	0.09
Left mandibular ramus length	34.48 (5.26)	37.05 (5.30)	0.05
Anteroposterior pole of right condyle	8.15 (1.15)	7.88 (1.1)	0.32
Anteroposterior pole of left condyle	7.72 (1.01)	8.08 (1.2)	0.19
Midlateral pole of left condyle	16.04 (3.41)	15.94 (3.53)	0.90
Midlateral pole of right condyle	15.82 (2.41)	15.57 (3.73)	0.73
Mandibular Deviation (mm)	6.31 (3.46)	4.11 (2.20)	0.003 **

Average (standard deviation); ** *p* < 0.005.

**Table 5:** Diagnostic performance for two criteria (cut-off values: MD = 6 mm and MD = 4) to classify UCH as active/inactive based upon MD.

Diagnostic Performance	Cut-Off Value MD = 6mm	Cut-Off Value MD = 4mm
TP	22	30
TN	23	17
FP	8	14
FN	18	10
Sensitivity	55.00%	75.00%
Specificity	74.19%	54.84%
Correct classification	63.8%	66.2%
PPV	73.33%	68.18%
NPV	56.10%	62.96%
LR (+)	2.13	1.66
LR (−)	0.61	0.46

MD: mandibular deviation; TP (true positive); TN (true negative); FP (false positive); FN (false negative); PPV (positive predictive value); NPV (negative predictive value); LR+ (likelihood ratio positive); LR− (likelihood ratio negative).
